# PSAPP mice exhibit regionally selective reductions in gliosis and plaque deposition in response to S100B ablation

**DOI:** 10.1186/1742-2094-7-78

**Published:** 2010-11-16

**Authors:** Emily Roltsch, Leigh Holcomb, Keith A Young, Alexander Marks, Danna B Zimmer

**Affiliations:** 1 Department of Veterinary Pathobiology, Texas A&M University, College Station, TX, USA; 2 Psychiatry and Behavioral Science, Texas A & M Health Science Center, College of Medicine, Temple, TX, USA; 3 Central Texas Veterans Health Care System Neuropsychiatry Research Program, Temple, TX, USA; 4 Banting & Best Department of Medical Research, University of Toronto, Ontario, Canada

## Abstract

**Background:**

Numerous studies have reported that increased expression of S100B, an intracellular Ca^2+ ^receptor protein and secreted neuropeptide, exacerbates Alzheimer's disease (AD) pathology. However, the ability of S100B inhibitors to prevent/reverse AD histopathology remains controversial. This study examines the effect of S100B ablation on *in vivo *plaque load, gliosis and dystrophic neurons.

**Methods:**

Because S100B-specific inhibitors are not available, genetic ablation was used to inhibit S100B function in the PSAPP AD mouse model. The PSAPP/S100B^-/- ^line was generated by crossing PSAPP double transgenic males with S100B^-/- ^females and maintained as PSAPP/S100B^+/- ^crosses. Congo red staining was used to quantify plaque load, plaque number and plaque size in 6 month old PSAPP and PSAPP/S100B^-/- ^littermates. The microglial marker Iba1 and astrocytic marker glial fibrillary acidic protein (GFAP) were used to quantify gliosis. Dystrophic neurons were detected with the phospho-tau antibody AT8. S100B immunohistochemistry was used to assess the spatial distribution of S100B in the PSAPP line.

**Results:**

PSAPP/S100B^-/- ^mice exhibited a regionally selective decrease in cortical but not hippocampal plaque load when compared to PSAPP littermates. This regionally selective reduction in plaque load was accompanied by decreases in plaque number, GFAP-positive astrocytes, Iba1-positive microglia and phospho-tau positive dystrophic neurons. These effects were not attributable to regional variability in the distribution of S100B. Hippocampal and cortical S100B immunoreactivity in PSAPP mice was associated with plaques and co-localized with astrocytes and microglia.

**Conclusions:**

Collectively, these data support S100B inhibition as a novel strategy for reducing cortical plaque load, gliosis and neuronal dysfunction in AD and suggest that both extracellular as well as intracellular S100B contribute to AD histopathology.

## Background

S100B, a member of the S100 protein family, is expressed predominantly in astrocytes and functions as both an intracellular Ca^2+ ^receptor and an extracellular neuropeptide [1-3]. The term S100 to refers to the solubility of these 10,000 molecular weight proteins in saturated ammonium sulfate [4]. S100 proteins are distinguished from other members of the S100/calmodulin/troponin superfamily of EF-hand Ca^2+ ^binding proteins by their 3 D structure and highly conserved 14 amino acid Ca^2+ ^binding loop [5]. Upon binding Ca^2+^, S100 proteins undergo a conformational change which exposes a hydrophobic patch necessary for interacting with numerous intra- and extracellular protein targets and subsequent exertion of their biological effects [5,6]. Over 20 intracellular targets have been reported for S100B suggesting that it regulates a large number of diverse cellular processes, including energy metabolism, cell proliferation, cytoskeletal organization, Ca^2+ ^homeostasis and signal transduction pathways. The extracellular effects of S100B are concentration dependent; nanomolar S100B levels beneficially promote neuronal survival while micromolar levels detrimentally promote apoptosis [7-9]. S100B's extracellular effects are thought to be mediated by the receptor for advanced glycation end products (RAGE) [7,8]. S100B release/secretion is regulated by forskolin, lysophosphatidic acid, serotonin, glutamate, IL-6β, metabolites and the neurotoxic Aβ peptide [10-14] as well as being gender- and age-dependent [15]. Increased S100B levels are associated with a variety of neurological disorders including Alzheimer's disease (AD), multiple sclerosis, amyotrophic lateral sclerosis, schizophrenia, epilepsy, alcoholism, drug abuse, hypoxia and traumatic brain injury [1-3,16,17].

Altered S100B function is associated with AD pathobiology. The clinical presentation and pathology of early- and late-onset AD include early disturbances in Ca^2+ ^homeostasis followed by inflammation, neurodegeneration, senile plaques comprised of aggregated amyloid β (Aβ) peptide, intracellular neurofibrillary tangles comprised of aggregated hyperphosphorylated tau, and ultimately cognitive dysfunction [18-21]. In human autopsy specimens, the highest levels of S100B expression are observed in the most severely affected regions and S100B associates with plaques [3,22,23]. Serum/CSF S100B levels inversely correlate with cognitive function, i.e patients with lower S100B levels exhibit lower Clinical Dementia Rating scores and higher Mini-Mental State Examination scores [24]. In addition, the rs2300403 single nucleotide polymorphism (SNP) in the S100B gene is associated with low cognitive performance, dementia and AD [25]. While the cellular events/molecular mechanisms whereby S100B contributes to AD pathobiology have not yet been elucidated, S100B has been reported to regulate Aβ biogenesis, amyloid precursor protein expression/processing and tau hyperphosphorylation [26-28]. In turn, the Aβ peptide increases S100B levels [29] resulting in a positive feedback loop. Thus, S100B may be a key contributor to a detrimental "cytokine cycle" that drives the progression of AD [2,3,8,16,30].

*In vivo *studies in genetically modified mouse models have yielded conflicting results regarding the contribution of increased S100B expression to AD pathology. Transgenic TghuS100B mice express 4-5 fold more S100B protein [31] and exhibit increased hippocampal gliosis with no change in plaque load upon hippocampal Aβ infusion when compared to non-transgenic controls [32]. However, TghuS100B/Tg2576 mice exhibit increased plaque load/gliosis in the hippocampus as well as the cortex when compared to Tg2576 mice [26]. The mechanism(s) responsible for the differential effects of increased S100B expression on hippocampal pathology in the two AD models have not been elucidated. Pharmacological inhibition and genetic ablation have also produced contradictory results. Treatment of Tg2576 mice with arundic acid, an inhibitor of S100B expression (40-45% decrease), reduces plaque load/gliosis in the hippocampus and cortex [33]. Surprisingly, S100B ablation has no effect on hippocampal plaque load, gliosis or dystrophic neurons in an Aβ infusion model [32]. Thus, the ability of S100B inhibitors to prevent/reverse AD histopathology is not completely understood.

While specific inhibitors that block the interaction of S100B with its target proteins are under development, currently available compounds do not cross the blood-brain barrier and cannot be used to inhibit CNS S100B [34]. Therefore, this study uses an *in vivo *genetic approach which recapitulates the entire spectrum of S100 function (detrimental, beneficial, intracellular, and extracellular) to ascertain the net effect of S100B ablation on AD histopathology in the PSAPP AD mouse line. Although no AD mouse model exhibits all aspects of the human disease, the PSAPP double transgenic (APP_K670NM671L_/PS-1_M146L_) line has a rapid disease onset and mimics many facets of the human disease including plaque deposition, dystrophic neurites, glial activation, and memory deficits [35-38]. PSAPP/S100B knockout mice exhibited a regionally selective decrease in cortical but not hippocampal plaque load. Reductions in plaque load were accompanied by decreases in plaque number, GFAP-positive astrocytes, Iba1-positive microglia and phospho-tau positive neurons. Finally, S100B immunoreactivity in cortex and hippocampus of PSAPP mice was plaque associated and co-localized with astrocytes/microglia. These results suggest that secreted and intracellular forms of S100B contribute to AD pathology and that pharmacological strategies which selectively block S100B action in the CNS may be effective in treating AD.

## Methods

### PSAPP X S100B Knockout Mice

The PSAPP double transgenic line was generated by crossing the Tg2576 line ("Swedish" APP_K670N/M671L _mutation) with the 6.2 line (PS-1_M146L_) [35-38]. The S100B^-/- ^line has been described previously [39]. The PSAPP/S100B^-/- ^line was generated by crossing PSAPP double transgenic males with S100B^-/- ^females and subsequent interbreeding of the PSAPP/S100B^+/- ^heterozygous offspring (PSAPP/S100B^+/- ^X PSAPP/S100B^+/-^). To control for changes in genetic background, all experiments used PSAPP/S100B^+/+ ^and PSAPP/S100B^-/- ^littermates. Procedures involving animals were approved by the Texas A & M University Institutional Animal Care and Use Committee and comply with the *NIH Guide for the Care and Use of Laboratory Animals*.

For genotyping, amplification of a 500 bp product using PCR primers for the mouse β-casein gene (forward primer 5' GAT GTG CTC CAG GCT AAA GTT 3' and reverse primer 5' AGA AAC GGA ATG TTG TGG AGT 3') was used to assess genomic DNA quality. The PS-1 and APP transgenes were detected as previously described [36]. Amplification of 250 bp band (forward primer 5' GCA AAG AAC AGG GTA GAA AAC ATG AAA AAC G 3'; reverse primer 5' GCC ATT CAA ACT AAT ATC CAG AAG CAA CCC 3') was used to detect the wild-type S100B allele. PCR programs contained a 5 minute denaturation step at 95°C; followed by thirty cycles consisting 1 minute at 94°C, 2 minutes at 60°C, and 3 minutes at 72°C; as well as a final 7 minute extension step at 72°C.

### Sample Acquisition/Processing

Brains were removed from anesthetized animals, rinsed in phosphate buffered saline (PBS) and fixed in 4% (wt/vol) paraformaldehyde in PBS for 30 minutes. Sagittal slices, 2 mm in thickness, were prepared using an acrylic brain matrix (Ted Pella, Redding CA) and post-fixed for an additional 30 minutes. Slices were then permeabilized in 2 mM MgCl_2_, 0.01% (wt/vol) sodium deoxycholate, 0.02% (vol/vol) Nonidet P-40 in 100 mM sodium phosphate buffer pH 7.5 for 36-48 hours. After post-fixation in 10% buffered formalin for 16 hours, tissues were embedded in paraffin and 5 micron sagittal sections were mounted on glass slides for subsequent staining. This processing procedure, originally developed for visualization of β-galactosidase reporter gene activity in transgenic mouse tissues, provides optimum S100B antibody specificity/sensitivity without compromising the detection of other antigens.

### Immunohistochemical and Congo red staining

To minimize variability, sections from experimental and control groups were processed simultaneously. Consecutive slides (2/animal) each containing sections at Allen Brain Atlas Sagittal Levels 8 and 17 were depariffinized and rehydrated to distilled water. For Congo red staining, slides were incubated in 0.02 M NaOH in 80% ethanol saturated with NaCl for 20 minutes followed by a 30 minute incubation in 0.2% (wt/vol) Congo Red (Cat. 150711, MP Biomedicals, LLC, Solon, OH) in 0.02 M NaOH in 80% ethanol saturated with NaCl, dehydration and mounting. Immunostaining was performed on a DAKO autostainer (Dako, Carpinteria, CA) using a biotin-free polymer detection kit (MACH 2, Biocare Medical, Walnut Creek, CA) and conditions recommended by the primary antibody manufacturer. Primary antibodies for immunohistochemistry included a mouse monoclonal S100B antibody (1-1000 dilution of Z0311 Dako); rabbit polyclonal GFAP antibody (1-1000 dilution of Z0334, Dako); mouse monoclonal Iba1antibody (1-300 dilution of SC-32725, Santa Cruz Biotechnology, Santa Cruz, CA); and mouse monoclonal Ser202/Thr205 phosphorylated tau antibody (1-20 dilution of MN1020, Pierce Chemical Co., Rockford, IL). For immunofluorescence microscopy, the anti-Iba1 antibody was diluted 1-10, the anti-GFAP antibody 1-100, and the anti-S100B antibody 1-50 (612377 from BD Transduction Laboratories, San Jose, CA). Secondary antibodies included an Alexa Fluor 546 donkey anti-rabbit (1-200 dilution of A10040, Molecular Probes, Carlsbad, CA); Alexa Fluor 488 rabbit anti-mouse (1-200 dilution of A11059, Molecular Probes); and Alexa Fluor 546 donkey anti-mouse (1-200 dilution of A10036, Molecular Probes).

For quantification, digital images were captured at 10× magnification on an Olympus IX70 Imaging System using a single exposure setting as follows: the entire hippocampus (2 images); the visual (1 image), somatosensory (1 image) and somatomotor (1 image) cortex as well as representative areas of the cerebellum and olfactory bulb. Images were converted to gray scale and the threshold intensity was set to the intensity observed in areas without tissue. Image J software (NIH Image, Bethesda, MD) was used to quantify positive pixels, plaque size, plaque number and total area. Plaque load and immunoreactivity were defined as the % area, i.e. the area of positive pixels/total pixels × 100. The data were expressed as the mean ± SEM (n = 8 for PSAPP and n = 6 for PSAPP/S100B^-/-^). An independent samples t-test (SPSS Inc., Chicago, IL) was used to determine the significance (p < 0.05) of measured differences between the two genotypes. Pearson's Correlation Coefficient and scatter plots of the mean hippocampal and cortical GFAP/Iba1 burden versus plaque load for each animal were used to determine the relationship between plaque load and astrocytosis/microgliosis.

Images for colocalization experiments were obtained on a Zeiss 510 META NLO laser scanning microscope. The following settings were used for fluorophore detection: DAPI excitation G 365, Dichroic FT 395, BP 445/50; for Alexa 488, exciter BP470/20, Dichroic FT 493, Emission BP 505-530; and Alexa 568, Exciter BP560/40, Dichroic FT 585, Emission BP 630/75. Images were collected, corrected for background and bleedthrough (reference images) and colocalization (overlap coefficient) of GFAP/S100B and Iba1/S100B determined using the LSM software.

## Results

### S100B ablation reduces cortical but not hippocampal plaque load

**Figure 1:**
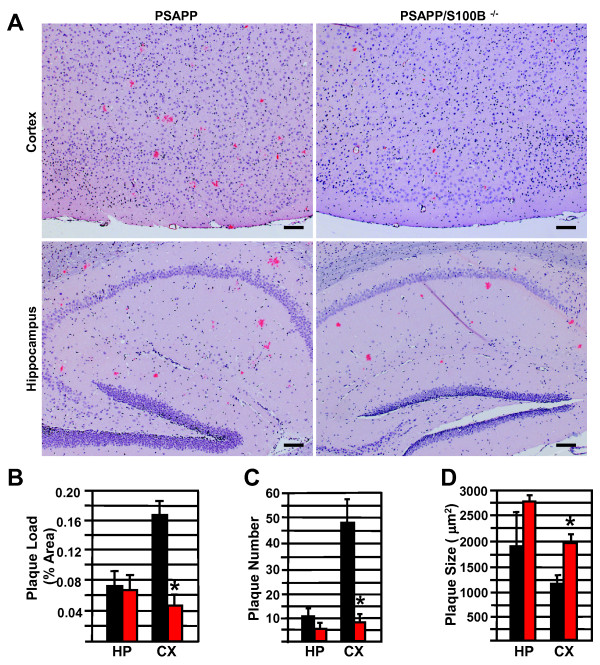
**S100B ablation reduces cortical, but not hippocampal plaque load**. Panel A contains representative micrographs of parasagittal cortical/hippocampal sections from 6 month old PSAPP and PSAPP/S100B^-/- ^mice stained with Congo red (pink) and hematoxylin (blue) to visualize plaques and nuclei, respectively (bars = 50 μm). The histograms depict the mean congophilic plaque load (Panel B), plaque number (Panel C), and plaque size (Panel D) ± SEM in PSAPP (black bars, n = 8), and PSAPP/S100B^-/- ^(red bars, n = 6) mice. Asterisks denote p ≤ 0.05 when compared to the PSAPP group.

To determine if S100B ablation altered amyloidogenesis, plaque load was quantified in PSAPP/S100B knockout and PSAPP mice. Congo red stained fibrillar plaques were observed in the hippocampus and cortex of 6 month old PSAPP/S100B^-/- ^and PSAPP mice (Figure 1A). In fact, congophilic plaque load in the control PSAPP littermates was indistinguishable from previous reports [35-38]. Furthermore, hippocampal plaque load in the two genotypes was indistinguishable: 0.070 ± 0.020 and 0.075 ± 0.022 percent area in the PSAPP/S100B^-/- ^and PSAPP controls, respectively (Figure 1B). Hippocampal plaque size and number were also similar in the two genotypes (Figure 1C and 1D). In contrast, there was a 3-fold reduction in cortical plaque load in PSAPP/S100B^-/- ^mice (0.050 ± 0.016 percent area) when compared to PSAPP mice (0.168 ± 0.016 percent area) (Figure 1B). This decrease in cortical plaque load was accompanied by an 5-fold reduction in plaque number (47.78 ± 9.42 vs 8.78 ± 2.07) and a slight increase in plaque size (1162 ± 141 vs 1955 ± 196 μm^2^) (Figure 1C and 1D). In summary, this is the first demonstration that S100B ablation selectively reduces cortical plaque load.

### S100B ablation decreases cortical, but not hippocampal, gliosis

**Figure 2:**
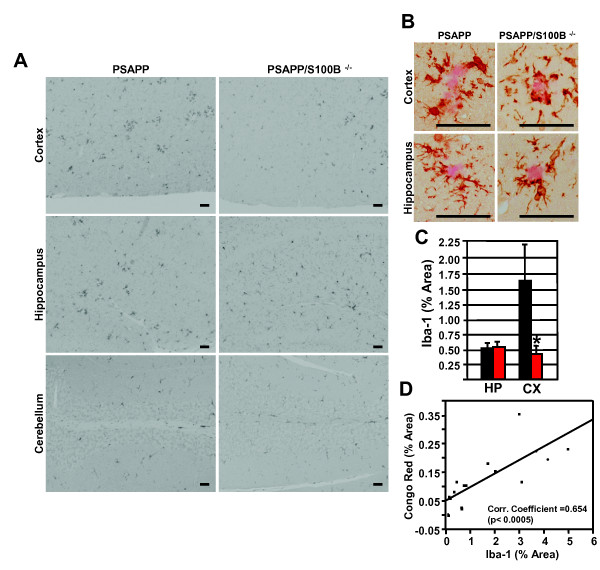
**S100B ablation reduces cortical microgliosis**. Panel A contains representative photomicrographs of parasagittal cortical/hippocampal sections from 6 month old PSAPP and PSAPP/S100B^-/- ^mice stained with the microglial marker Iba1. Panel B contains high magnification images from consecutive sections co-stained Iba1 (brown) and Congo red (pink). Scale bars = 50 μm. The histograms in Panel C depict the mean Iba1 positive area ± SEM in PSAPP (black bars, n = 8), and PSAPP/S100B^-/- ^(red bars, n = 6), mice. Asterisks denote p ≤ 0.05 when compared to the PSAPP group. Panel D is a scatter plot of the mean Iba1 burden versus plaque load for each animal (cortex and hippocampus).

**Figure 3:**
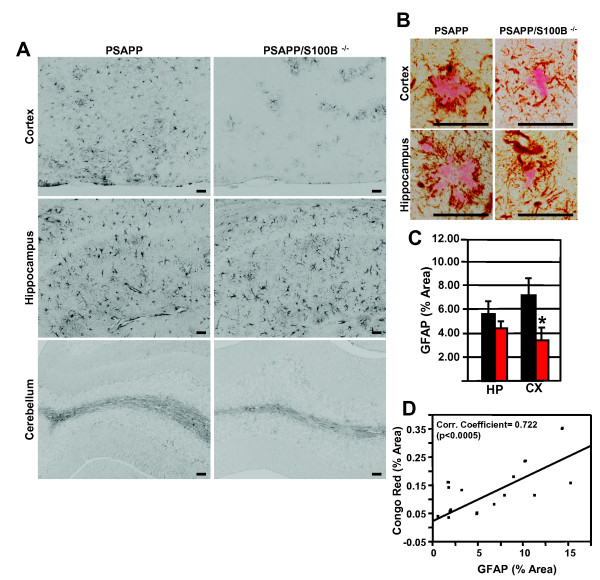
**S100B ablation reduces cortical astrocytosis**. Panel A contains representative photomicrographs of parasagittal cortical/hippocampal sections from 6 month old PSAPP and PSAPP/S100B^-/- ^mice shown stained with the astrocytic marker a GFAP. Panel B contains high magnification images from consecutive sections co-stained Iba1 (brown) and Congo red (pink). Scale bars = 50 μm. The histogram in Panel C illustrates the mean GFAP positive area ± SEM in PSAPP (black bars, n = 8), and PSAPP/S100B^-/- ^(red bars, n = 6) mice. Asterisks denote p ≤ 0.05 when compared to the PSAPP group. Panel D is a scatter plot of the mean GFAP burden versus plaque load for each animal (cortex and hippocampus).

S100B's deleterious effects in the central nervous system have been attributed to reactive gliosis (astrocytosis and microgliosis) [23,26,32,33]. The microglial marker Iba1 and astrocytic marker GFAP were used to determine if S100B ablation in PSAPP mice also reduced gliosis. Plaque-associated Iba1 staining of small cell bodies and long processes was observed in the cortex and hippocampus of PSAPP and PSAPP/S100B^-/- ^mice (Figure 2A and 2B). This staining pattern was similar to previous reports for AD mouse models (Tg2576) [26,33]. Hippocampal Iba1 burden in the two genotypes was similar (0.51 ± 0.12 vs. 0.51 ± 0.10 percent area) (Figure 2C). However, cortical Iba1 burden was 4-fold less (0.45 ± 0.09 vs 1.69 ± 0.49 percent area) in PSAPP/S100B^-/- ^mice when compared to PSAPP control mice (Figure 2C). In both genotypes, the Iba1 burden was similar in non-plaque containing regions such as the cerebellum (Figure 2A) and olfactory bulb (data not shown). Furthermore, there was a direct correlation between Iba1 burden and plaque load (Pearson's Correlation Coefficient 0.654, p < 0.0005) (Figure 2D). Hippocampal/cortical plaque-associated GFAP positive astrocytes were also observed in both genotypes (Figure 3A and 3B) and the staining pattern (somata and processes) was indistinguishable from previous reports for AD mouse models [26,33]. Hippocampal GFAP burden was similar (4.53 ± 0.67 vs. 5.52 ± 1.35 percent area) while cortical GFAP burden was 2-fold less (3.56 ± 0.92 vs. 7.26 ± 1.40 percent area) in PSAPP/S100B^-/- ^mice when compared to PSAPP control mice (Figure 3C). In addition, GFAP burden in non-plaque containing regions such as the cerebellum (Figure 3A) and olfactory bulb (data not shown) was similar in the two genotypes. Like Iba1, there was a direct correlation between GFAP burden and plaque load (Pearson's Correlation Coefficient 0.722 p < 0.0005) (Figure 3D). Collectively, these findings demonstrate that S100B ablation results in regionally selective decreases in microgliosis and astrocytosis that directly correlate with plaque load.

### Effects of S100B ablation on dystrophic neurons

**Figure 4:**
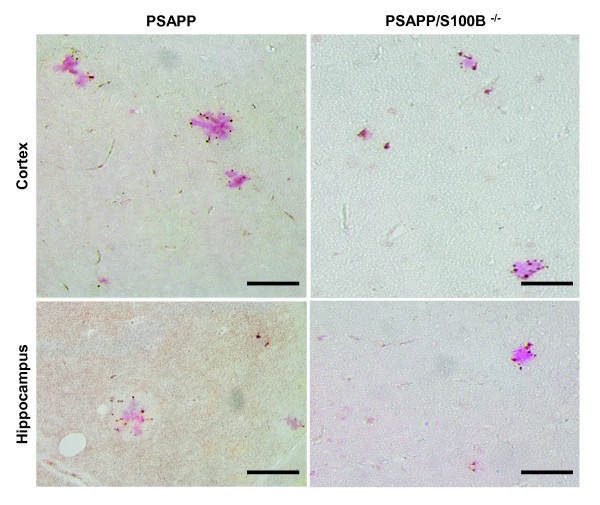
**Phospho-tau staining in PSAPP and PSAPP/S100B^-/- ^mice**. Representative photomicrographs of parasagittal cortical and hippocampal sections from 6 month old PSAPP and PSAPP/S100B^-/- ^mice co-stained with the AT8 antibody which recognizes tau phosphorylated at Ser202/Thr205 and Congo red to visualize plaque associated dystrophic neurites. Scale bars = 50 μm.

Although the correlation between plaque load and cognitive function remains controversial, decreases in plaque load are commonly accompanied by reductions in dystrophic neurons/neurites and improvements in cognitive function. In fact, changes in phospho-tau levels/staining are used to detect dystrophic neurons in PSAPP and Tg2576 mice despite the fact that these models do not develop tangles [40-42]. Therefore, the AT8 phospho-tau antibody, which detects phospho-Ser202/Thr205, was used to ascertain the effect of S100B ablation on dystrophic neurons. The hippocampal and cortical phospho-tau staining patterns in both genotypes was indistinguishable from previous reports: punctate plaque-associated staining (Figure 4). These results demonstrate that S100B ablation does not prevent the development of plaque-associated dystrophic neurons. However, as predicted by the plaque load results, PSAPP/S100B^-/- ^mice exhibited fewer cortical but similar numbers of hippocampal phospho-tau foci/plaques when compared to PSAPP mice (Figure 4).

### S100B colocalizes with hippocampal as well as cortical astrocytes, microglia, and plaques

**Figure 5:**
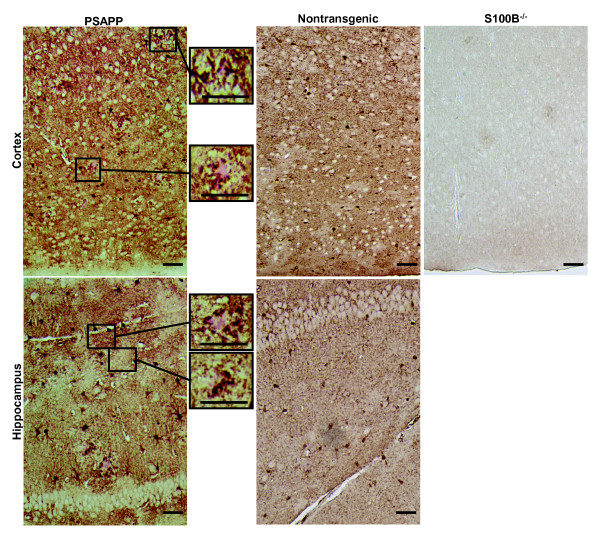
**S100B distribution in the PSAPP AD mouse model**. Representative photomicrographs of parasagittal cortical and hippocampal sections from 6 month old PSAPP, non-transgenic littermates or S100B^-/- ^mice stained with an S100B antibody (brown). Sections from PSAPP mice were co-stained with Congo red (pink) which is visible in the high magnification insets. Scale bars = 50 μm.

**Figure 6:**
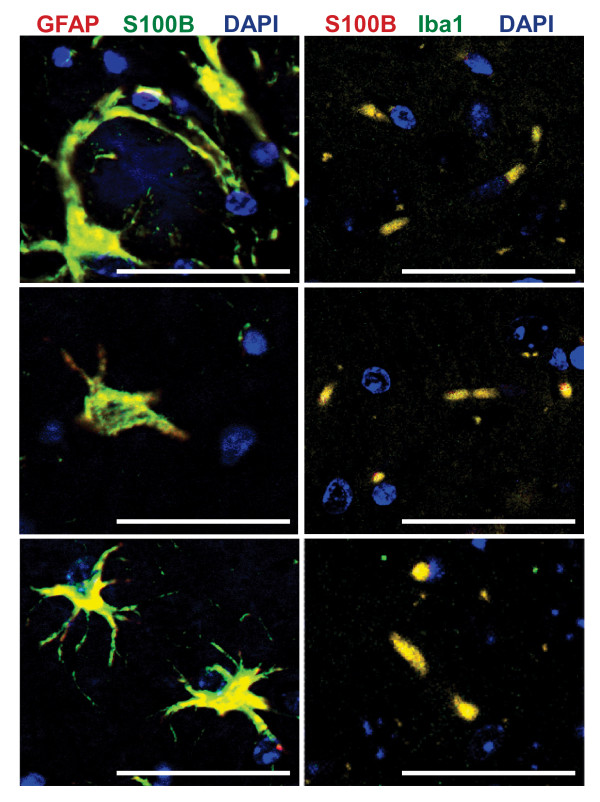
**S100B staining co-localizes with microglia and astrocytes**. Representative fluorescent micrographs of parasagittal cortical sections from 6 month old PSAPP mice co-stained with a GFAP (red) and an S100B (green) antibody or an Iba1 (green) and an S100B (red) antibody. Yellow denotes co-localization of S100B with astrocytes or microglia. Scale bars = 50 μm.

In human autopsy specimens and the Tg2576 mouse model, S100B staining is associated with astrocytes and plaques [22,23,26]. In addition, in human AD the highest levels of S100B are observed in the most severely affected regions [23]. Therefore, S100B immunohistochemistry was used to determine if differences in S100B distribution were responsible for the regionally selective effects of S100B ablation on histopathology in PSAPP mice. In nontransgenic mice, intense staining of astrocytic cell bodies/processes and diffuse cytoplasmic/extracellular S100B staining was observed in the hippocampus and cortex (Figure 5). PSAPP mice exhibited a similar staining pattern as well as punctate plaque-associated staining (Figure 5). The increased staining intensity in PSAPP mice when compared to nontransgenic mice is consistent with previous reports of increased S100B expression in AD [1-3]. Sections from PSAPP/S100B^-/- ^mice exhibited no detectable staining (Figure 5) indicating that plaque-associated and diffuse cytoplasmic/extracellular staining were not attributable to non-specific antibody binding and/or high background. The S100B staining pattern observed in PSAPP mice was similar to staining patterns observed in the Tg2576 AD mouse model [33] and human autopsy specimens [43]. The cellular distribution of S100B in PSAPP mice was confirmed by double immunofluorescence staining with astrocytic (GFAP, overlap coefficient 0.8083 ± 0.0149) and microglial (Iba1, overlap coefficient 0.8476 ± 0.0356) markers (Figure 6). Collectively, these findings suggest that both intracellular and extracellular forms of S100B may contribute to AD histopathology. In conclusion, the regionally selective effects of S100B ablation on histopathology are most likely attributable to regional differences in the cellular processes that are regulated by S100B and not differences in S100B expression/distribution.

## Discussion

This study definitively demonstrates that S100B ablation/inhibition reduces AD pathology. PSAPP/S100B knockout mice exhibited a regionally selective decrease in cortical but not hippocampal plaque load that was accompanied by reductions in astrocytosis, microgliosis and dystrophic neurons. These regionally selective effects were not attributable to variations in the S100B distribution; cortical and hippocampal S100B staining patterns were indistinguishable in PSAPP mice. Finally, in PSAPP mice S100B immunoreactivity was associated with plaques and colocalized with astrocytes as well as microglia suggesting that both intracellular and extracellular S100B contribute to AD histopathology. Interestingly, other studies have reported regionally selectively effects of S100B ablation on Ca^2+ ^handling, synaptic plasticity, kainate-induced gamma amplitudes and BDNF (brain-derived neurotrophic factor) levels [44-47]. Ascertaining the molecular mechanisms responsible for S100B's selective effects on AD histopathology and other processes may provide new insights regarding the events that contribute to the non-uniform progression of AD [48-50].

These findings clarify inconsistencies in the literature regarding S100B's contribution to AD histopathology. Genetic ablation (this study), pharmacological inhibition [33] and genetic overexpression [26] approaches consistently indicate that decreases in S100B reduce AD histopathology in the cortex. The larger effects observed with genetic ablation may be due to maximal inhibition; pharmacological inhibition reduces S100B levels by 40-50% and genetic overexpression increases S100B levels by 30% [26,33]. In all S100B^-/- ^mice hippocampal plaque load remains unchanged regardless of the mechanism used to induce plaque deposition, i.e. Aβ infusion [32] or overexpression of mutant proteins (APP and PS-1) in transgenic mice (this study). However, pharmacological inhibition of S100B synthesis with arundic acid in mice that overexpress mutant APP (TG2576 line) reduces hippocampal plaque load/gliosis [33] and overexpression of S100B in the same model increases hippocampal plaque load/gliosis [26]. These differential effects may be due to alternative mechanisms of action for arundic acid [51], upregulation of compensatory mechanisms in knockout models, gain of function in overexpression models, differences in the AD mouse models and/or variations the ages of the animals.

A consistent finding in this and previous studies is a direct correlation between changes in plaque load and gliosis/inflammation in response to alterations in S100B expression. It is unclear, however, whether changes in plaque load are the cause or the result of changes in gliosis. Furthermore, it is unclear how these histopathological changes impact cognitive function. Microglia are an essential component of the inflammatory response and exist in many forms [52,53]. They beneficially phagocytose plaques and suppress inflammation as well as detrimentally promote inflammation and neuronal cell death [54-56]. Detailed analyses of microglial/glial phenotypes in PSAPP/S100B^-/- ^mice will be instrumental in identifying S100B-regulated events that contribute to AD pathology and in discerning the relationship between plaques and inflammation. Behavioral data are not available for any of the S100B/AD mouse models. Strengthened synaptic plasticity and enhanced spatial memory in S100B^-/- ^mice [44] suggest that PSAPP/S100B^-/- ^mice will exhibit improved cognitive function. This hypothesis is supported by the inverse correlation of serum/CSF S100B levels and direct correlation of the rs2300403 SNP in the S100B gene with low cognitive performance, dementia and AD [24,25]. Experiments are underway to determine if pharmacological inhibition of S100B expression and/or interaction of S100B with its target proteins will improve cognitive function in AD and/or other neurological disorders.

S100B's plaque association and co-localization with cells (microglia/astrocytes) in this and previous studies [22,23,33] suggest that both intracellular and extracellular S100B contribute to AD pathology. Inhibition of intracellular S100B would be predicted to reduce Aβ-induced spontaneous calcium transients [29], decrease inflammatory cytokine release [2,3,30] and prevent Aβ-induced increases in S100B levels [10]. Inhibition of extracellular S100B would be predicted to alter Ca^2+ ^handling, synaptic plasticity long-term potentiation, neuronal apoptosis and/or BDNF levels [39,43-47,57,58]. Decreased extracellular S100B-RAGE/scavenger receptor signaling in glia, neurons and/or endothelial cells [7,9,59-64] could also impact APP synthesis, APP processing and/or tau phosphorylation (GSK3β, cdk5 and/or PKA pathways) [18-21]. In fact, intracellular/extracellular S100B may link dysregulation of Ca^2+ ^homeostasis with AD pathobiology and/or serve as a common upstream regulator of both tau phosphorylation/neurofibrillary tangles and Aβ production/plaque deposition [18-21]. While astrocytes, microglia and oligodendrocytes are the most logical source of S100B, peripheral tissues such as adipose cannot be excluded [65-67]. Defining the source of and mechanisms of release/secretion for S100B will be important steps in delineating the S100B-regulated processes that contribute to AD histopathology.

## Conclusions

Collectively, these data definitively demonstrate that S100B ablation reduces plaque load, gliosis and dystrophic neurons in the cortex but not the hippocampus. If this reduction in histopathology can be demonstrated to positivity impact cognitive changes related to AD, then additional impetus to the search for new therapeutic interventions targeted at S100B will be provided. The development of effective pharmacological strategies for modulating S100B function in patients will also require quantifying the contribution of extracellular versus intracellular forms, identifying the S100B-regulated target proteins/cellular processes, and ascertaining the contribution of the five other S100 family members implicated in AD, S100A1, S100A6, S100A7, S100A9, and S100A12 [1,68-70]. Finally, the beneficial effects of S100B ablation/inhibition may extend to other neurological disorders that involve dysregulation of glial cell calcium homeostasis [71,72].

## Competing interests

The authors declare that they have no competing interests.

## Authors' contributions

LH and KY provided the PSAPP mouse line. AM provided the S100B knockout mouse line. DZ and LH conceived the study and developed the PSAPP/S100B knockout mouse line. ER performed the experiments and participated in the writing of the manuscript as partial fulfillment of the requirements for the PhD degree. DZ supervised the data collection/analyses and drafted the manuscript. All authors read and approved the final manuscript.
